# Supplementary material for: Phytochemical Profiling and Antioxidant Activities of the Most Favored Ready-to-Use Thai Curries, Pad-Ka-Proa (Spicy Basil Leaves) and Massaman
Source: Foods. 2024 Feb 14;13(4):582. doi: 10.3390/foods13040582 (PMC10887624; doi:10.3390/foods13040582)
Supplement: Supplementary file 1 [file foods-13-00582-s001.zip › Supplementary data paper2.pdf]

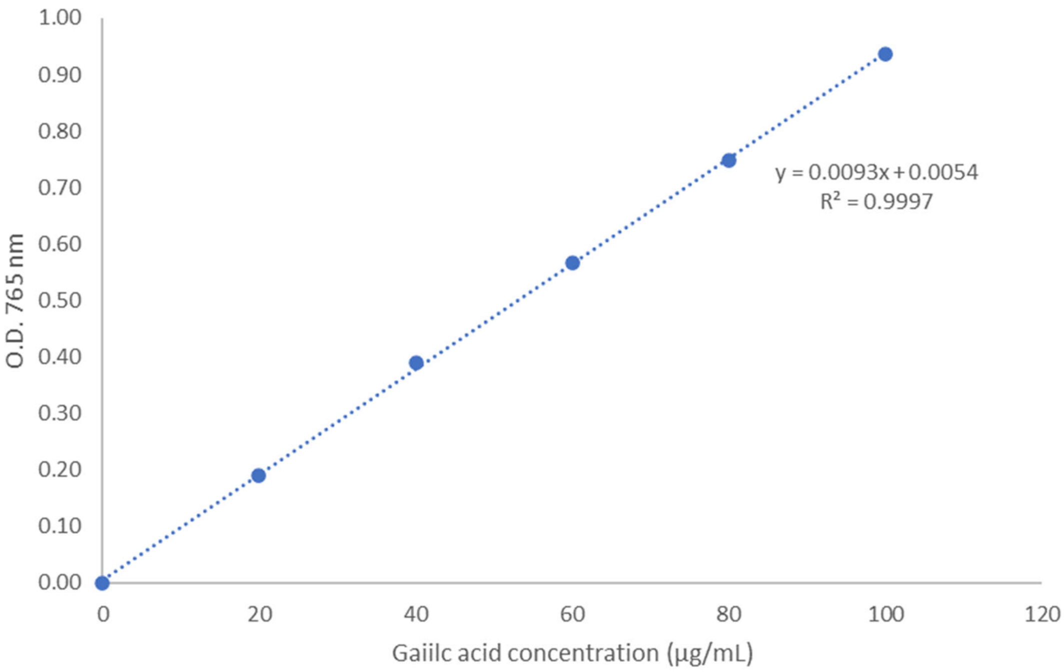

Figure S1 Standard curve of total phenolic content (TPC) with gallic acid agent used as a standard.

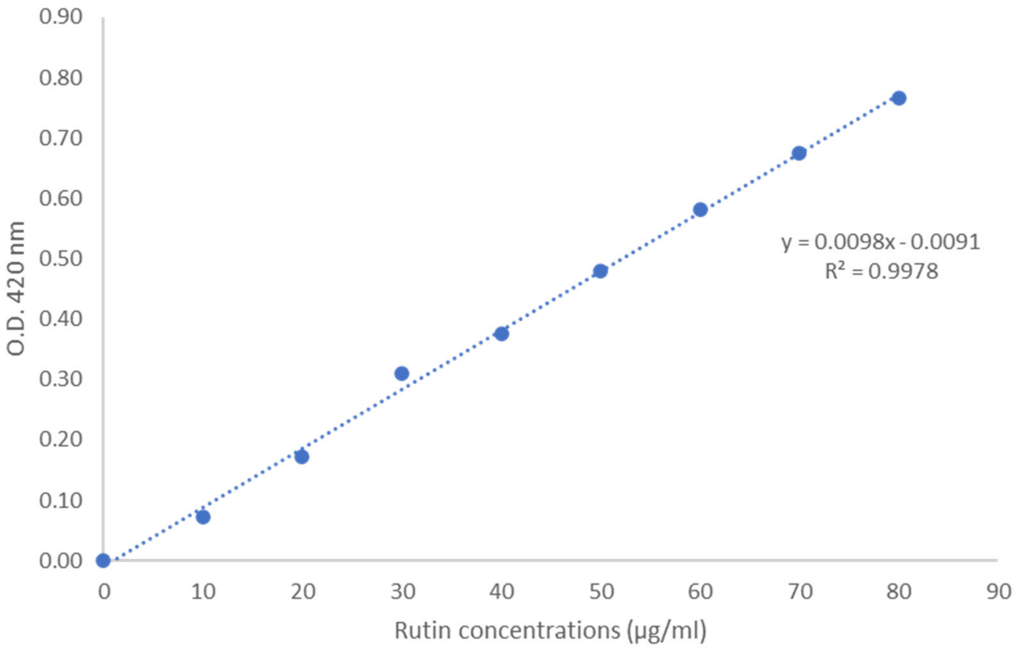

Figure S2 Standard curve of total flavonoid content (TFC) with rutin agent used as a standard.

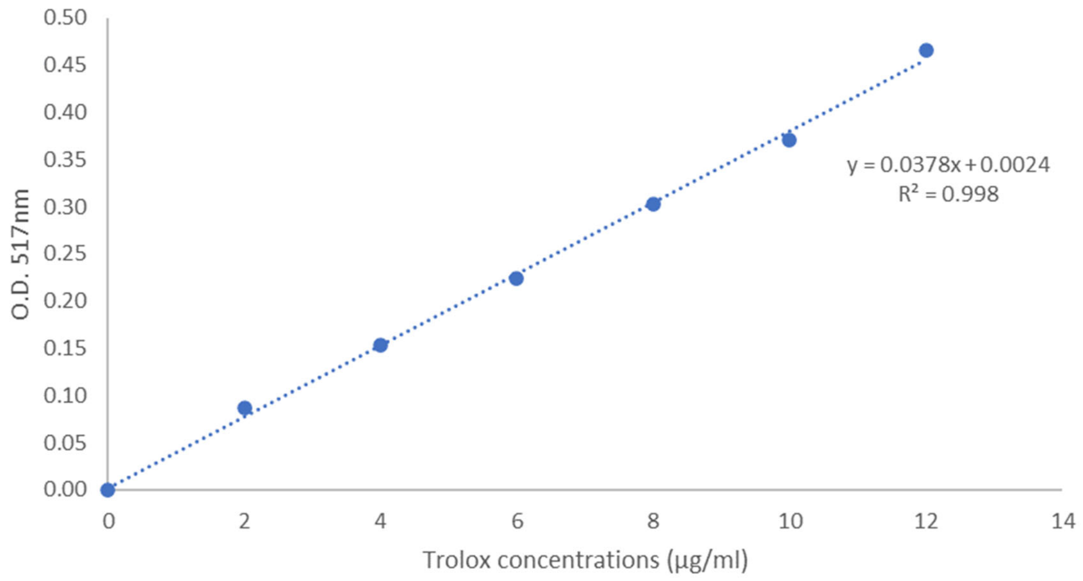

Figure S3 Standard curve of DPPH assay with Trolox agent used as a standard.

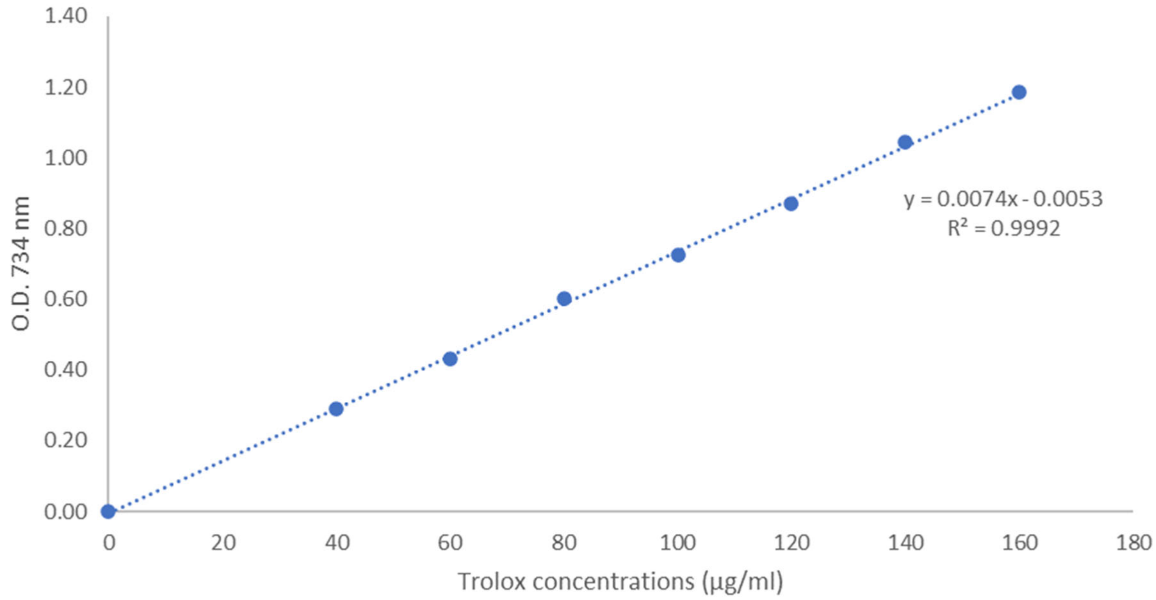

Figure S4 Standard curve of ABTS assay with Trolox agent used as a standard.

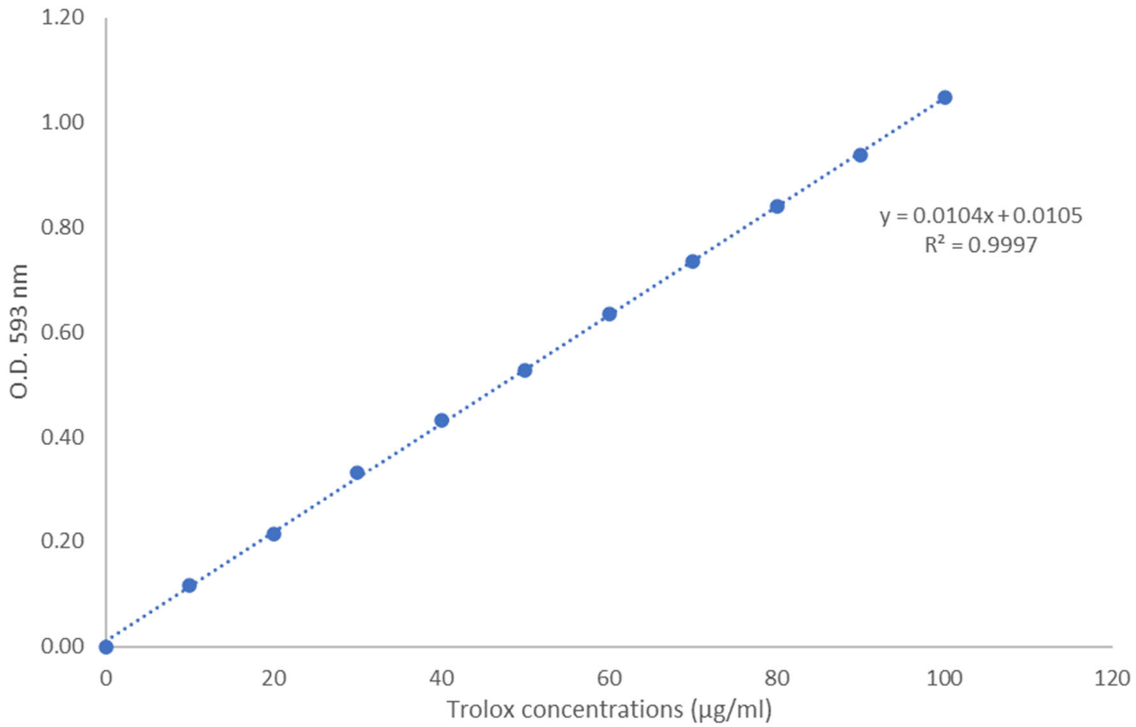

Figure S5 Standard curve of FRAP assay with Trolox agent used as a standard.

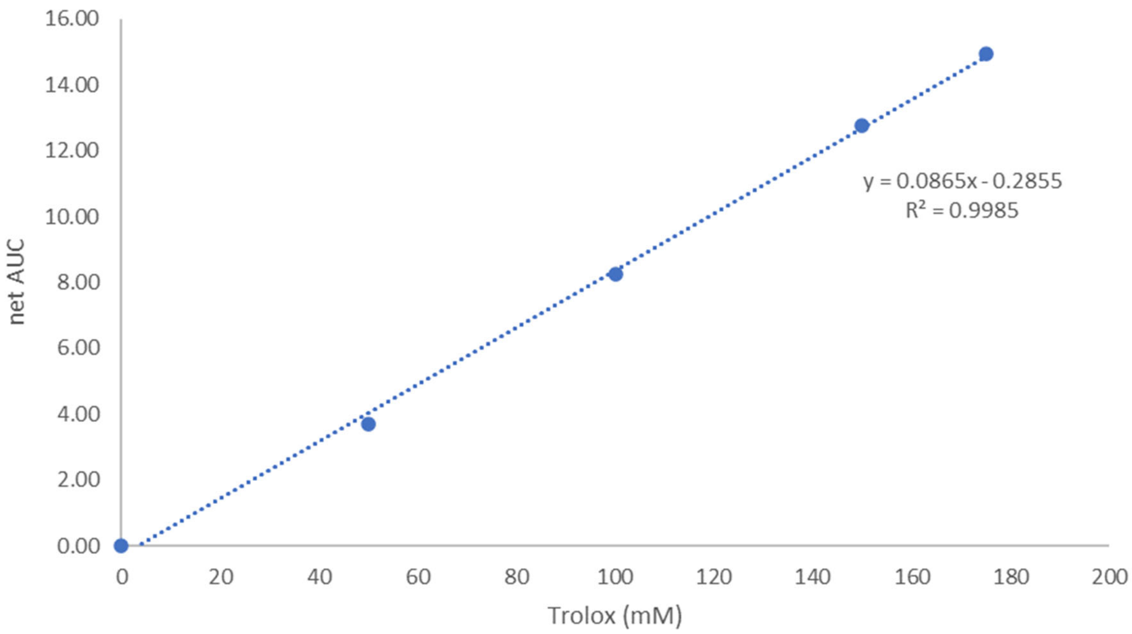

Figure S6 Standard curve of ORAC assay with Trolox agent used as a standard.

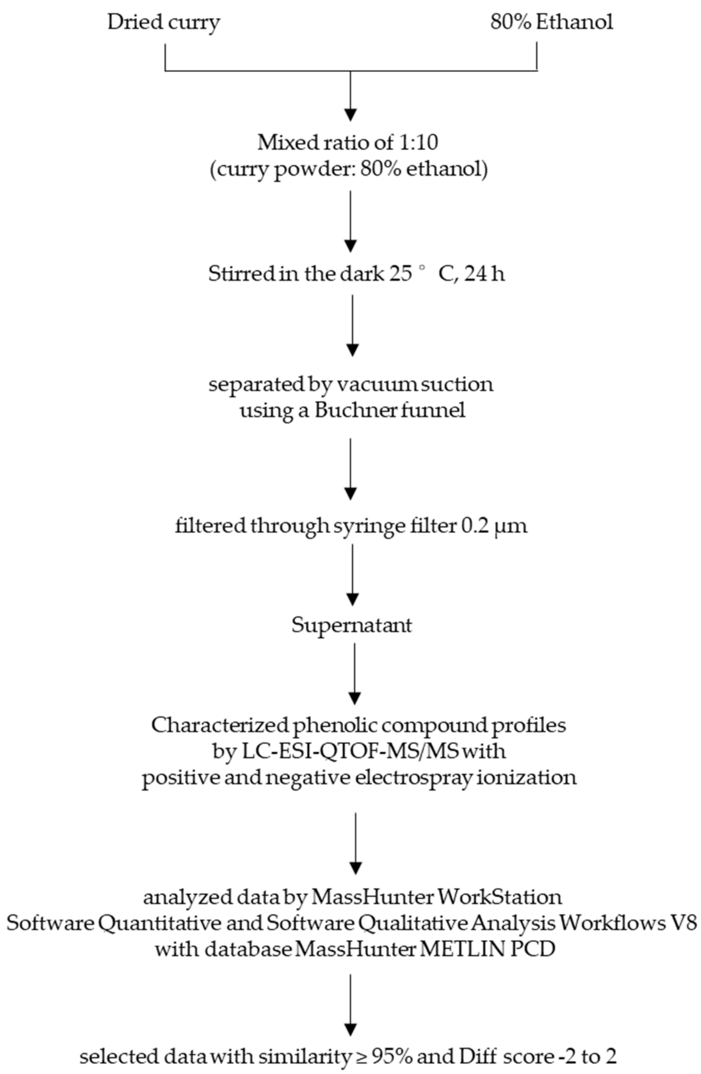

Figure S7 Diagram of characterization of phenolic profiles by LC-ESI-QTOF-MS/MS.

Table S1 Correlation coefficients of total phenolic content, total flavonoid content, and antioxidant activities.

**Variables**	**TPC**	**TFC**	**DPPH**	**ABTS**	**FRAP**
TFC	0.974**				
DPPH	0.996**	0.971**			
ABTS	0.994**	0.967**	0.994**		
FRAP	0.994**	0.966**	0.993**	0.995**	
ORAC	0.974**	0.898*	0.981**	0.981**	0.985**

** Correlation is significant at $p<0.01$ and * Correlation is significant at $p<0.05$
